# New York County Veterinary Medical Association

**Published:** 1896-02

**Authors:** Robert W. Ellis

**Affiliations:** Secretary


### NEW YORK COUNTY VETERINARY MEDICAL ASSOCIATION.

THE regular meeting of this Association was held on Tuesday, January 7, 1896, at 8.45 P.M., with the President, Dr. Huidekoper, in the chair.

The Secretary being delayed, Dr. Neher was requested to act as Secretary pro tem., and proceeded by calling the roll, to which the following members responded: Drs. Amling, Bretherton, C. C. Cattanach, J. S. Cattanach, J. J. Cattanach, Delaney, Dixon, Ferster, Foy, Gill, Hanson, Huidekoper, Neher, O'Shea, and Sherwood.

After which the President appointed to act as Board of Censors H. D. Gill, H. D. Hanson, J. S. Cattanach, J. E. Ryder, and J. H. Ferster, and as a Committee on Legislation Arthur O'Shea, J. E. Ryder, S. S. Field, Herbert Neher, and F. W. Turner. There being no papers, Dr. Sherwood spoke a few words on mange, and also agreed to read a paper at the next meeting.

Dr. Ferster then reported a "Case of Azoturia with Partial Paralysis in the Hind Leg as a Sequela," and questions if cases will recover. Dr. Amling reported two similar cases.

Dr. Neher reported "Six Cases of Azoturia," with similar symptoms in forelegs. At this point the Secretary arrived and took his chair, and a general discussion on azoturia and its treatment continued.

Dr. Ferster asked the chair for his after-treatment in azoturia when any of the above sequelæ remained, and he recommended full doses of potassii iodidum for a time and turning the patient out.

Drs. Neher, O'Shea, and J. S. Cattanach gave their views on treatment in different stages of the disease.

The President then asked the meeting their pleasure in regard to having the minutes of the previous meeting read, now that the books were on hand, and it was moved and seconded that it be deferred until the next meeting.

The question of "Tetanus and its Treatment" next came up and was discussed generally by the members.

Dr. O'Shea reported recovery after the use of nicotine in full doses on the tongue, followed by digitalis and whiskey.

Dr. J. S. Cattanach reported a case in which recovery followed one injection of 3 grains of morphine. Dr. Ellis reported a case in which all the characteristic symptoms were well marked, but not far advanced, in which recovery followed one injection of 25 c.c. tetanus antitoxin.

Dr. Gill mentioned a similar case in which recovery followed an injection of diphtheria antitoxin. It was moved and seconded that the discussion close. Carried. It was moved and seconded that the meeting adjourn. Carried.

ROBERT W. ELLIS, D.V.S.,
Secretary.

